# Biomarker and pathway analyses of urine metabolomics in dairy cows when corn stover replaces alfalfa hay

**DOI:** 10.1186/s40104-016-0107-7

**Published:** 2016-08-31

**Authors:** Huizeng Sun, Bing Wang, Jiakun Wang, Hongyun Liu, Jianxin Liu

**Affiliations:** Institute of Dairy Science, MoE Key Laboratory of Molecular Animal Nutrition, College of Animal Sciences, Zhejiang University, Hangzhou, 310058 People’s Republic of China

**Keywords:** Biomarker, Dairy cow, Metabolomics, Pathway, Urine

## Abstract

**Background:**

Alfalfa hay and corn stover are different type of forages which can significantly impact a cow’s lactation performance, but the underlying metabolic mechanism has been poorly studied. We used biomarker and pathway analyses to characterize related biomarkers and pathways based on urine metabolomics data from different forage treatments. Urine was collected from 16 multiparous Holstein dairy cows fed alfalfa hay (AH, high-quality forage, *n* = 8) and corn stover (CS, low-quality forage, *n* = 8) respectively. Gas chromatography–time of flight/mass spectrometry (GC-TOF/MS) was performed to identify metabolites in urine and the metaboanalyst online platform was used to do biomarker and pathway analysis.

**Results:**

Hippuric acid (HUA) and N-methyl-glutamic (NML-Glu) indicated the most significant difference between the two diets, when statistically validated by biomarker analysis. HUA was also validated by standard compound quantitative method and showed significant higher concentration in CS group than AH group (2.8282 vs. 0.0005 mg/mL; *P* < 0.01). The significant negative correlation between milk yield and HUA (R^2^ = 0.459; *P* < 0.01) and significant positive correlation between milk yield and NML-Glu (R^2^ = 0.652; *P* < 0.01) were characterized. The pathway analysis revealed that these different metabolites were involved in 17 pathways including 7 influential pathways (pathway impact value > 0): Tyr metabolism, starch and sucrose metabolism, amino sugar and nucleotide sugar metabolism, galactose metabolism, Phe, Tyr and Try biosynthesis, purine metabolism, and glycerolipid metabolism. Based on the metabolome view map, the Phe, Tyr and Try biosynthesis pathway exhibited the highest impact value (0.50), and the Holm-Bonferroni multiple testing-based analysis revealed the most significant difference in the Tyr metabolism pathway (Holm *P* = 0.048).

**Conclusions:**

The identified HUA and NML-Glu may serve as potential biomarkers for discriminating CS and AH diets and could be used as candidates for milk yield related mechanistic investigations. Integrated network pathways associated with related metabolites provide a helpful perspective for discovering the effectiveness of forage quality in lactation performance and provides novel insights into developing strategies for better utilization of CS and other low-quality forage in China.

**Electronic supplementary material:**

The online version of this article (doi:10.1186/s40104-016-0107-7) contains supplementary material, which is available to authorized users.

## Background

Forage quality greatly affects dairy cow performance [1, 2]. Using traditional nutritional methods, it is difficult to discern metabolic alterations and characterize alterations in key metabolic pathways when dairy cows are fed diets differing in forage quality. Metabolomics is an effective means to explain the overall complex and essential changes in diverse biological systems and may be the sole technology that can detect these changes [3]. This is further improved by combining nuclear magnetic resonance or mass spectra (MS) based high-throughput identification methods and multivariate statistical analyses [4]. The metabolomics approach is a useful tool for elucidating the effects of diet on biofluid metabolite profiles in dietary intervention studies on dairy cows [5].

In previous work, we characterized certain results for common metabolites in four biofluids (rumen fluid, milk, serum, and urine) [6]. We concluded that a deep analysis of metabolites from a single representative biofluid should be used to generate specific biomarkers and pathways to distinguish the metabolic profiles of dairy cows fed different diets. Urine metabolites result from global metabolism within the body and are easily affected by physiological, dietary and environmental interventions. As an easily collected and stored biofluid, urine has been widely used in human metabolomics to diagnose disease and serve as an early warning in preclinical stages [7]. Urine metabolomics may be used to capture most of the small molecular compounds in urine and identify those that significantly differ to further characterize metabolic pathways that may differ among different dietary treatments [8]. This will provide insight into identifying biomarkers and understanding the physiological processes associated with performance differences [9].

In the current study, biomarker and related pathway analysis was performed using the aforementioned urine metabolomics raw data under different forage treatments to evaluate the effects of forage quality on dairy cow metabolism. This approach can discern valid biomarkers and pathways and to understand the mechanism underlying forage-related nutrition in dairy cows.

## Methods

### Experimental design

The experimental procedures were approved by the Animal Care Committee at the Zhejiang University (Hangzhou, China) and were in accordance with the university’s guidelines for animal research.

Sixteen multiparous (3.6 ± 1.8 parity) Holstein cows were randomly assigned to 2 groups according to BW and milk yield as described by Sun et al. [6]. Both groups were offered consisting of 55 % concentrate mixture and 15 % corn silage with different forages (Additional file 1: Table S1): (1) alfalfa (AH), containing 23 % alfalfa hay and 7 % Chinese wild rye hay, and (2) corn stover (CS), containing 30 % corn stover instead of alfalfa hay and Chinese wild rye hay. The diet was formulated to meet or exceed the net energy requirement for cows with milk production at 30 kg/d [10]. Feed was offered *ad libitum* to allow for at least 5 to 10 % orts. The experiment lasted for 65 d long with the first 15 d were used for adaption. Cows were housed in a tie-stall barn with free access to drinking water and were fed and milked 3 times daily at 0630, 1330 and 1930 h.

### Sample collection and metabolite measurement

At the end of the experiment, urine samples (15 mL) were collected using vulval stimulation between 0500 and 0630 h. Each sample was immediately frozen in a liquid nitrogen container to minimize metabolite degradation. After thawing and centrifugation at 6,000 × g at 4 °C for 15 min, samples were stored in 1.5 mL centrifuge tubes at −80 °C for further analyses. The methods and procedures for identifying specific metabolomics, including metabolite extraction, derivatization, GC-TOF/MS identification and data pretreatment, are described elsewhere [6]. L-2-chlorophenylalanine was used as a internal standard and bistrifluoroacetamide (containing 1 % TCMS, v/v) as a derivated reagent. Agilent 7890 GC system equipped with a Pegasus 4D TOFMS (LECO, St. Joseph, MI, USA) was installed with a DB-5MS capillary column (0.25 μm film thickness, 30 m × 250 μm inner diameter). Helium served as the carrier gas and a front inlet purge flow of 3 mL/min was utilized. Temperature procedure was as follows: the initial temperature was kept at 80 °C for 0.2 min, increased to 180 °C at a rate of 10 °C/min, to 240 °C at a rate of 5 °C/min, and further to 290 °C at a rate of 20 °C/min; the column was then maintained for 11 min. The injection, transfer line, and ion source temperatures were 280, 245, and 220 °C, respectively. The MS data were acquired at a rate of 100 spectra/s after a solvent delay of 492 s with a mass-to-charge ratio (m/z) range of 20 to 600 in full-scan mode.

### Hierarchical cluster analysis

In a previous study [6], 31 significantly different metabolites were identified in the urine. The relative concentration of these significantly different metabolites was incorporated into an online analysis platform (Metaboanalyst 3.0, http://www.metaboanalyst.ca/) for a hierarchical cluster analysis (HCA), which is a widely used data summary analysis tool to merge similar groups of points into visualization tree. Each sample began as a separate cluster, and the algorithm combined the samples until each sample belonged to one cluster. The HCA with Euclidean distance similarity measures and an average linkage method was used to explore clustering patterns among the samples and metabolites in urine. The expression patterns and a heat map of each variable were generated using an average linkage hierarchical clustering program. High-correlation samples were positioned near the top of the dendrogram, and highly similar metabolites were assigned near the left of the dendrogram. Other multivariate statistic analysis (principal component analysis (PCA) and partial least squares discriminant analysis (PLS-DA)) were also performed using Metabolyst 3.0. The PCA was used to visualize the dataset and display similarities and differences. PLS-DA was performed to sharpen the separation between groups of observations, and to understand which variables carry the class separating information.

### Biomarker analysis and validation

Biomarker analysis was performed using statistical and mathematical modeling methods to select the minimum number of metabolites to represent and explain difference between 2 treatments. A subset of metabolites was manually selected to construct a classifier. One or more metabolites can be selected based on their difference between 2 groups (e.g., VIP value, *P* value, fold change). The classifier was then evaluated as a biomarker by analyzing the receiver operating characteristic (ROC), including the ROC view, predicted class probabilities, and cross validation (CV) prediction. The ROC analyses were based on a linear SVM algorithm. To produce a smooth ROC curve, 100 cross validations were performed, and results averaged to generate the plot. Using a probability view, a figure was generated to show the average predicted class probabilities for each sample among the 100 cross-validations. The classification boundary was located at the center for a balanced subsampling approach. Primarily, the effective sensitivity and specificity was evaluated based on the value of the area under the ROC curve (AUC), the sample distribution in the probability view, and CV prediction accuracy.

Biomarkers were validated by standard compound quantitative method using GC-TOF/MS. A standard curve was generated using a 4 mg/mL stock solution of hippuric acid (98 % assay; Sgima-Aldrich). The equipment used is previously described. However the temperature procedure differed and as follows: the initial temperature was kept at 50 °C for 1 min, then raised to 300 °C at a rate of 20 °C/min, then kept for 6.5 min at 300 °C. The injection, transfer line, and ion source temperatures were 280, 280, and 220 °C, respectively. The energy was −70 eV in electron impact mode. The mass spectrometry data were acquired in full-scan mode with the m/z range of 30–600 at a rate of 20 spectra/s after a solvent delay of 4.93 min.

### Related pathway characterization

Pathway characterization is used to expand metabolomic analyses to understand the systems-level effects of metabolites [11]. The relative concentrations of 31 significantly different metabolites were imported into Metaboanalyst to generate the metabolome view, which integrates pathway enrichment analysis and pathway topology analysis. In doing so subtle but consistent changes among a group of related compounds may be identified [12]. A global test was used in pathway enrichment analysis to determine whether a group of metabolites in one specific pathway is differentially expressed, which shifts individual metabolite analysis to a group of metabolites analysis [13]. Pathway topological analysis was based on the relative betweenness and out of degree centrality measures of a metabolite in a given metabolic network to calculate the metabolites importance [14]. The pathway impact was calculated as the sum of the importance measures of the matched metabolites normalized by the importance of all metabolites in each pathway [15]. The differential response in metabolites between the two groups were further identified using online databases, including the Kyoto Encyclopedia of Genes and Genomes (KEGG), Bovine Metabolome Database, PubChem Compound, Chemical Entities of Biological Interest and Chemical Abstracts Service. Each different metabolite was crosslisted with the pathways in the KEGG; the top altered pathways were then identified and constructed according to the potential functional analysis.

## Results

### Metabolic profiles and hierarchical cluster analysis

The PCA and PLS-DA analyses of the GC-TOF/MS metabolic profiles clearly show separated clusters in the 2D-PCA score plot between the AH and CS groups (Additional file 1: Figure S1), suggesting that the GC-TOF/MS-based urine metabolomics model can be used to identify the difference between the two diets.

**Fig. 1:**
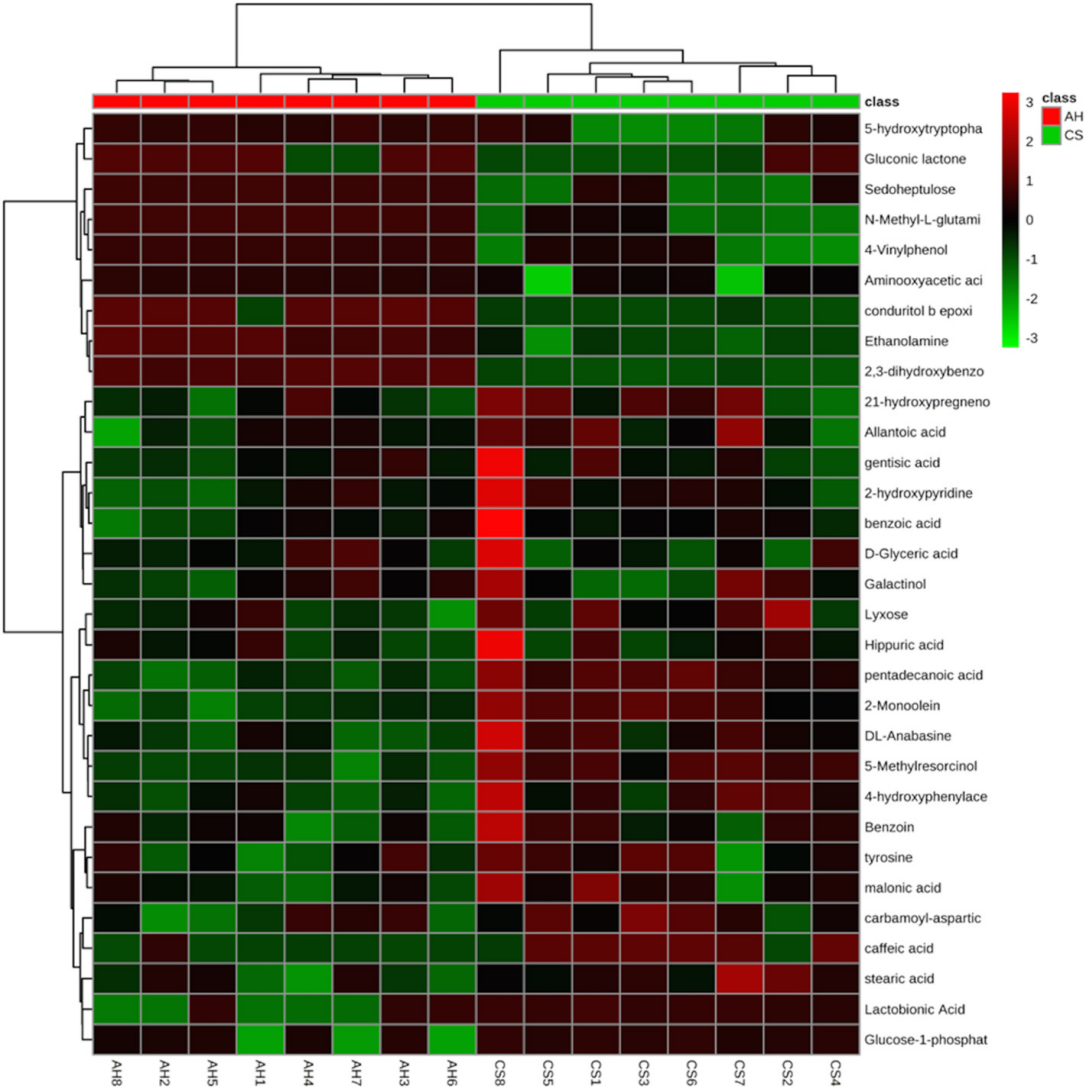
A hierarchical clustering analysis for the significantly different urine metabolites. The patterns in each row were determined using an average linkage hierarchical clustering program. The light blue boxes indicate an expression ratio less than the mean, and the dark red boxes denote an expression ratio greater than the mean. Tree clusters and their shorter Euclidean distances indicate higher similarities. Similarity between two metabolites is represented by branch height; thus, when a node is lower vertically, the subtree is more similar

The relative concentrations of 31 significantly different metabolites identified in the urine are shown in Fig. 1 with changes in the color intensity (from light blue to dark red) on the heat map. Different subclusters containing different numbers of samples in one diet fully clustered together, indicating a clear and strong difference between the AH and CS diets. Therefore, biomarker and pathway analyses based on these 31 significantly differently metabolites are credible.

### Analysis and validation of biomarker

**Fig. 2:**
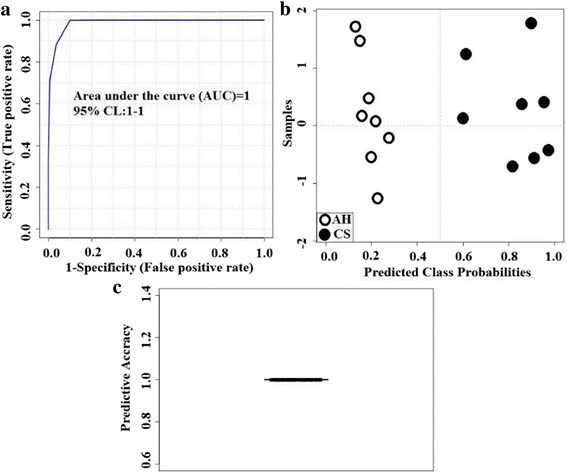
Biomarker analysis results (ROC view). Statistical method to evaluate treatment effectiveness using selected represented metabolites. **a** Probability view and **b** cross validation prediction of **c** 2 selected metabolites, hippuric acid and N-methyl-L-glutamic acid. CS = diet containing corn stover as the main forage; and AH = diet containing alfalfa as the main forage

Based on the individual AUC, fold change (FC), and p-value, hippuric acid and N-methyl-L-glutamic acid (NML-Glu) were selected as potential biomarkers because they exhibited the most significant difference between the diets (Additional file 1: Table S1). The biomarker analysis results for HUA and NML-Glu are shown in Fig. 2. The AUC was equal to 1 (Fig. 2a), and a clear separation and discrimination were observed between the CS and AH diets in the probability view (Fig. 2b). An AUC close to 1 indicates a more effective sensitivity and specificity. The average accuracy based on 100 cross validations was 1 (Fig. 2c) in this study. Having an average accuracy close to 1 indicates a more valid CV prediction. The concentration of HUA (mg/mL) in CS group was significantly higher than that in AH group (2.8282 vs. 0.0005; *P* < 0.01).

**Fig. 3:**
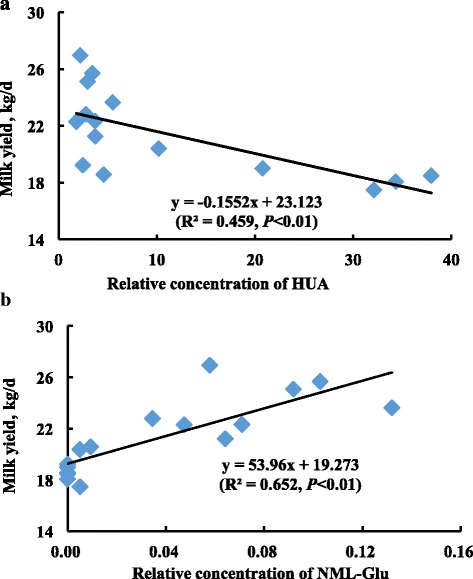
Correlation of milk yield and relative concentrations of hippuric acid (HUA), and N-methyl-L-glutamic acid (NML-Glu) in lactating cows, 2 panels were combined together in one image

A significant negative correlation was observed between milk yield and HUA concentration (R^2^ = 0.459, *P* < 0.01, Fig. 3a). On the contrary, milk yield was positively correlated with NML-Glu concentration (R^2^ = 0.652, *P* < 0.01, Fig. 3b), suggesting that these 2 metabolites may serve as candidates for future investigations into forage-related lactation mechanisms.

### Characterization and functional analysis of key metabolic pathways

Seventeen pathways were obtained when the significantly different metabolites were imported into KEGG. After enrichment and pathway topology analysis of the identified pathways, only 7 pathways showed an impact value at the comprehensive level (Table 1): Tyr metabolism; starch and sucrose metabolism; amino sugar and nucleotide sugar metabolism; galactose metabolism; Phe, Tyr and Try biosynthesis; purine metabolism; and glycerolipid metabolism. Among these 7 pathways, Phe, Tyr and Try biosynthesis exhibited the highest impact value (0.50). When the statistical *P* values were further adjusted via the Holm-Bonferroni method for multiple testing, only the Tyr metabolism exhibited significant differences (*P* = 0.048, Table 1).

**Table 1:** Results from the urine metabolomic pathway analyses in cows fed CS and AH diets^a^

Pathway	Hits^b^	*P* value	Home *P* ^c^	Impact value
Tyrosine metabolism	3	0.003	0.048	0.145
Starch and sucrose metabolism	1	0.030	0.416	0.150
Amino sugar and nucleotide sugar metabolism	1	0.030	0.416	0.086
Galactose metabolism	2	0.097	0.875	0.119
Phenylalanine, tyrosine and tryptophan biosynthesis	1	0.138	1.000	0.500
Purine metabolism	1	0.152	1.000	0.0002
Glycerolipid metabolism	1	0.967	1.000	0.105

^a^CS = diet containing corn stover as the main forage; and AH = diet containing alfalfa hay as the main forage

^b^Hits represents the number of metabolites in one pathway

^c^Home *P* indicates the statistical *P* values that were further adjusted using the Holm-Bonferroni method for multiple tests

**Fig. 4:**
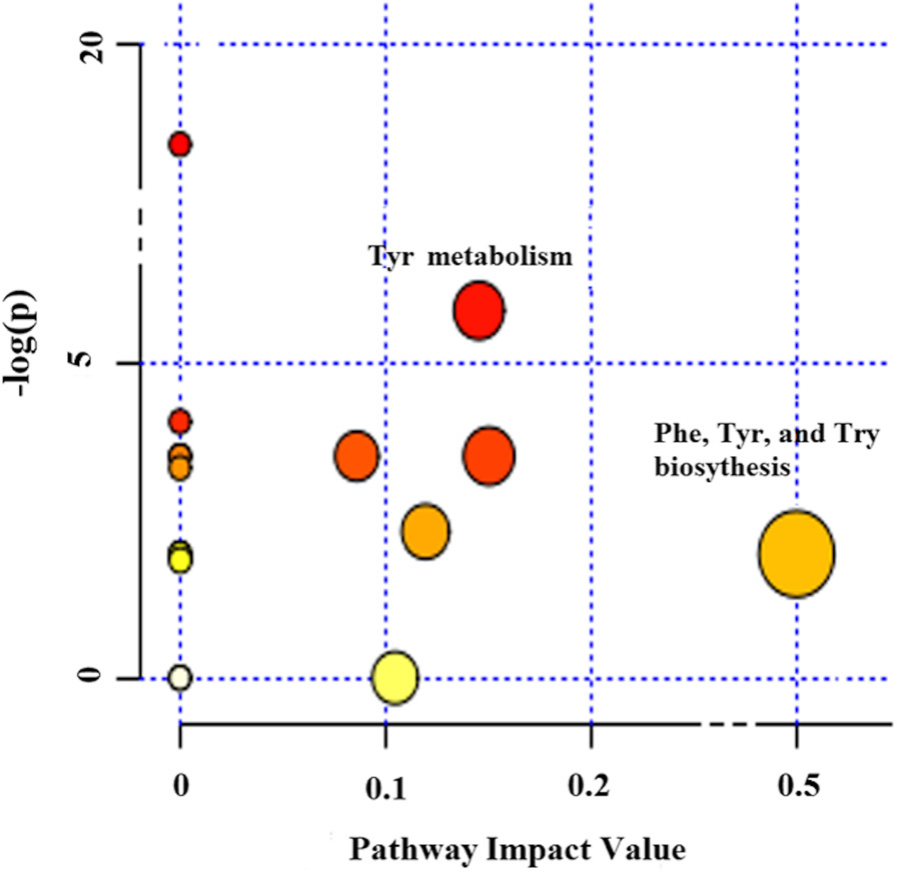
The metabolome view map of significant metabolic pathways characterized in urine for cows fed CS and AH. This figure aims to find pathways significant changed based on enrichment and topology analysis. The x-axis represents pathway enrichment, and the y-axis represents pathway impact. Larger sizes and darker colors represent greater pathway enrichment and higher pathway impact values, respectively. CS = diet containing corn stover as the main forage; and AH = diet containing alfalfa as the main forage

**Fig. 5:**
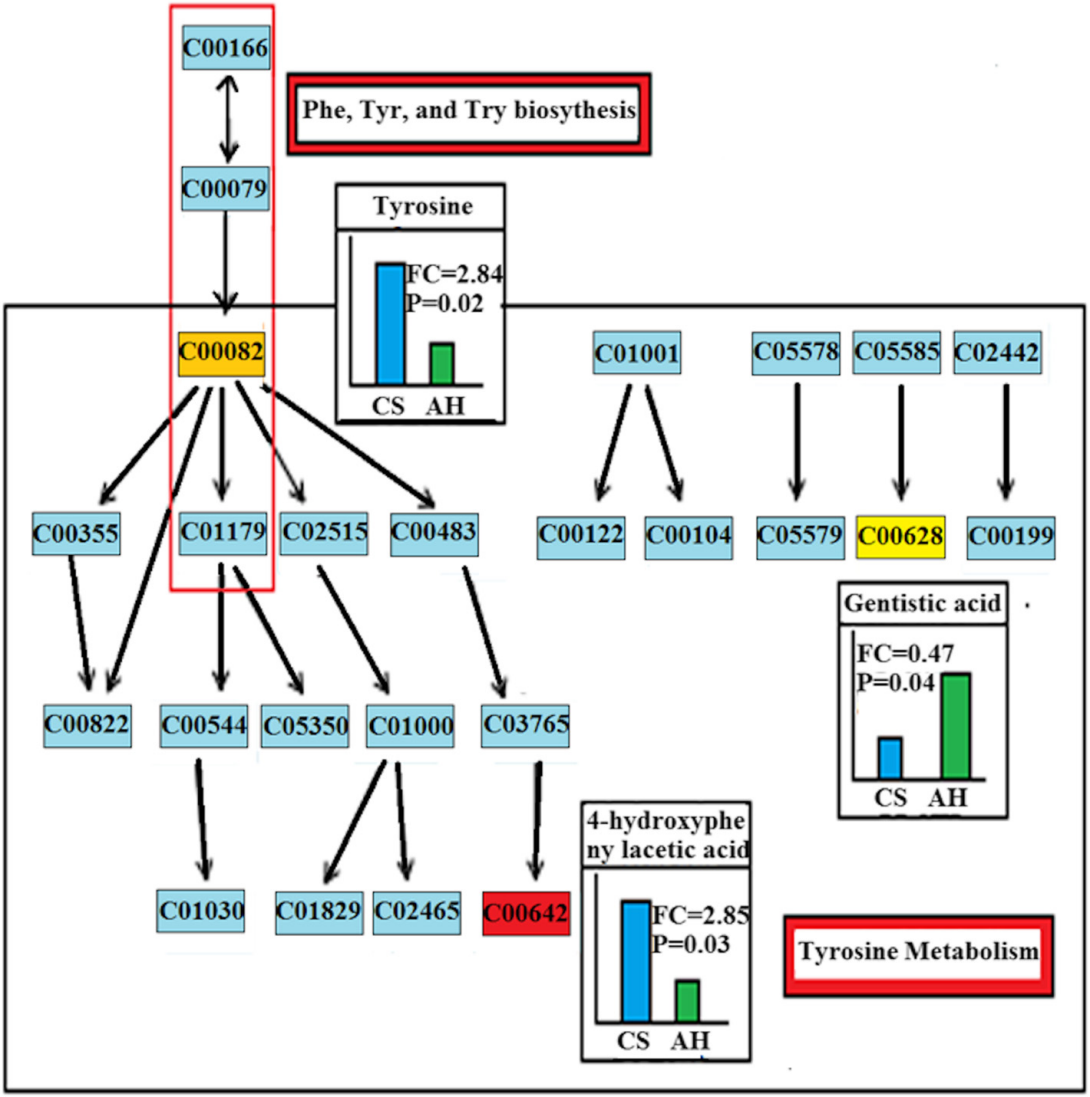
Key pathways altered by different forage quality and associated metabolites. The map was generated using the reference map from KEGG and consisted of entry number of metabolites and pathways. Metabolites that significantly different are marked with yellow or red background and display with column figure of relative concentration between 2 diets. Common metabolites are marked with blue background. CO represents the entry number of the compound. CS = diet containing corn stover as the main forage; and AH = diet containing alfalfa as the main forage

A comprehensive analysis of the *P* value and impact value showed that the pathways that differed the most were Tyr metabolism and Phe, Tyr and Try biosynthesis (Fig. 4). The integrated key pathways and involved metabolites are shown in Fig. 5. For Tyr metabolism pathway, 3 significantly different metabolites were characterized: Tyr (VIP = 1.38, *P* = 0.02; FC = 2.84), 4-Hydroxyphenylacetic acid (VIP = 1.35, *P* = 0.03; FC = 2.85) and gentisic acid (VIP = 1.21, *P* = 0.04; FC = 0.47). Tyr was also involved in Phe, Tyr and Try biosynthesis.

## Discussion

The key biomarkers, HUA and NML-Glu were successfully investigated using GC-TOF/MS combined with biomarker analyses and statistically validated. This suggests that these 2 metabolites may be used as biomarkers in urine from dairy cows when corn stover replaces alfalfa hay.

The HUA is a potential marker for determining the best type of goat feeding regimen [16]. HUA is an acyl glycine formed from conjugating benzoic acid with glycine in the liver [17] and is one of the five major nitrogenous components in urine of dairy cows [18]. HUA excretion is related to dietary concentrations of degradable phenolic acids [19]. Forage contains high levels of aromatic compounds, including HUA [20], which can be partly degraded in the rumen. HUA is absorbed in the rumen and intestines following an immediate conjugation before being transformed in the liver and excreted in urine [21]. In the current study, significant higher amount of HUA in CS group was validated by both statistical analysis and standard compound quantitative method. The greater HUA excretion in the CS-fed cows partly resulted in greater nitrogen loss compared with the cows fed AH. HUA excretion has been linked to lignin digestibility [22]. Further work is necessary to validate HUA as a biomarker in dairy cow nutrition.

NML-Glu is a Glu derivative with a methyl group added to the amino group and is an intermediate of methane metabolism [23]. It can also regenerate Glu through methylglutamate dehydrogenase. Glu is the major milk protein component [24], accounting for more than 20 % of milk protein and playing an important role in gluconeogenesis as a glucogenic precursor [25]. The function of NML-Glu in the glutamate pool warrants further investigation.

Based on the KEGG pathways, Tyr metabolism was one of the 13 AA metabolism pathways (KEGG map 00350). As shown in Fig. 5, the altered Tyr metabolism in dairy cows fed different quality forage mainly resulted in Tyr degradation. In contrast, the alterations of Phe, Tyr and Try biosynthesis pathway was attributed to Tyr biosynthesis. Tyr is an aromatic AA and a precursor for adrenalin, dopamine, norepinephrine and epinephrine, which play an important role in the sympathetic nervous system in animals [26]. In general, Tyr concentration in blood depends on dietary Tyr content [27]. Tyr is referred to as a semi-essential or conditionally indispensable AA because it only forms from Phe hydroxylation under certain condition [28]. During Tyr catabolism, the carboxyl carbon is almost immediately released as carbon dioxide, and the remaining portions of the Tyr molecule become either acetoacetate or fumarate [29], which can be used to synthesize AA or fatty acids.

Although Tyr is a non-essential AA, its synthesis within the body depends on Phe availability. Half of the Phe required for animals is used to produce Tyr. The requirement for Phe is reduced by approximately 50 % with a Tyr-rich diet [30]. The same feature was also identified in a previous study on dairy cows [31]. Phe is mainly hydroxylated to Tyr in the hepatic intracellular pool, which is irreplaceable in Tyr biosynthesis. Phe is an essential AA that must be supplied by dietary proteins. Once it has entered the body, Phe may follow one of three pathways: conversion to Tyr, incorporation into cellular proteins, or conversion to phenylpyruvic acid [32]. For dairy cows, valine and other branched-chain AA may reduce tyrosine absorption [33], which may limit conversion of essential AA, such as Phe, into proteins.

The integrated metabolic pathways contain interaction networks as well as related metabolites and provide information on nutritional intervention mechanisms [34]. This information extends beyond metabolic relevance and effects through the pathways and network analyses applied in the metabolomics analyses [35]. The detailed construction of the altered Tyr metabolism; the Phe, Tyr and Try biosynthesis pathways map; and the related metabolites suggest that the target pathways yield marked changes when forage quality varies. These biochemical changes may be used to understand the effects of different quality forages on lactation performance and provide insight for future exploration of mechanisms and cow nutrition.

## Conclusions

GC-TOF/MS technology and multivariate analyses were used to to show significant changes in urine metabolites and metabolic pathways between two diets containing AH or CS as the main forage. The identified compounds hippuric acid and N-methy-l-Glu may serve as a potential biomarker for discriminating between different forage quality. The Tyr metabolism and Phe, Tyr and Try biosynthesis pathways showed the most variation when corn stover replaced alfalfa hay. Insight into forage-related changes in physiology and metabolism may aid in developing strategies for better utilization of CS and other low-quality forages in China.

## Additional file

Additional file 1: Table S1. Ingredients of the experimental diets based on corn stover and alfalfa hay. **Table S2.** Identification of significantly different metabolites in urine between the CS and AH groups. **Figure S1.** The 2-D PCA score map (a) and 2-D PLS-DA score map (b) derived from the GC-TOF/MS metabolite profiles of urine for cows fed CS (red triangle) and AH (green plus). CS = dietcontaining corn stover as main forage; and AH = dietcontaining alfalfa and Chinese wild rye hay as main forage. (DOC 145 kb)

## Abbreviations

AH: Alfalfa hay
AUC: Area under the ROC curve
CS: Corn stover
CV: Cross validation
FC: Fold change
GC-TOF/MS: Gas chromatography–time of flight/mass spectrometry
Glu: Glutamic acid
HCA: Hierarchical cluster analysis
HUA: Hippuric acid
KEGG: Kyoto encyclopedia of genes and genomes
MS: Mass spectra
PCA: Principal component analysis
Phe: Phenlalanine
PLS-DA: Partial least squares discriminant analysis
ROC: Receiver operating characteristic
SVM: Support vector machine
Try: Tryptophane
Tyr: Tyrosine
VIP: Variable importance projection.
