# Supplementary material for: Biomarker and pathway analyses of urine metabolomics in dairy cows when corn stover replaces alfalfa hay
Source: J Anim Sci Biotechnol. 2016 Aug 31;7(1):49. doi: 10.1186/s40104-016-0107-7 (PMC5006375; doi:10.1186/s40104-016-0107-7)
Supplement: Additional file 1: Table S1. — Ingredients of the experimental diets based on corn stover and alfalfa hay. Table S2. Identification of significantly different metabolites in urine between the CS and AH groups. Figure S1. The 2-D PCA score map (a) and 2-D PLS-DA score map (b) derived from the GC-TOF/MS metabolite profiles of urine for cows fed CS (red triangle) and AH (green plus). CS = dietcontaining corn stover as main forage; and AH = dietcontaining alfalfa and Chinese wild rye hay as main forage. (DOC 145 kb) [file 40104_2016_107_MOESM1_ESM.doc]

**Supplemental Table S1.** Ingredients of the experimental diets based on corn stover and alfalfa hay.

| Item, % as a DM basis | Treatment***^a^*** | |
| --- | --- | --- |
|  | CS | AH |
| Ground corn grain | 27.0 | 27.0 |
| Wheat bran | 8.1 | 8.1 |
| Soybean meal | 7.5 | 7.5 |
| Cottonseed meal | 4.3 | 4.3 |
| Beet pulp | 2.2 | 4.1 |
| Corn silage | 15.0 | 15.0 |
| Alfalfa hat | 0.0 | 23.0 |
| Corn stover | 30.0 | 0 |
| Chinese wild rye hay | 0.0 | 7.0 |
| Urea | 1.0 | 1.0 |
| Premix*^b^* | 4.0 | 4.0 |
| Dicalcium phosphate | 0.1 | 0 |
| Limestone | 0.8 | 0 |

*^a^*CS = TMR containing corn stover as main forage; and AH = TMR containing alfalfa hay as main forage.

*^b^*Formulated to provide (per kg of DM) 500,000-700,000 IU of vitamin A; 140,000-170,000 IU of vitamin D_3_; 2,000-4,000 IU of vitamin E; 7,000-9,000 mg of Zn; 40-80 mg of Se; 180 mg of I; 1,400-2,500 mg of Fe; 15-30 mg of Co; 1,4000-2,500 mg of Mn; and 1,400-2,500 mg of Cu.

**Supplemental Table S2.** Identification of significantly different metabolites in urinebetween the CS and AH groups.

| Name | Similarity | AUC*^a^* | P-value | FC*^b^* |
| --- | --- | --- | --- | --- |
| N-Methyl-L-glutamic acid | 974 | 1 | <0.001 | 0.033 |
| Hippuric acid | 989 | 1 | 0.017 | 5.966 |
| Ethanolamine | 862 | 1 | 0.001 | 0.089 |
| 4-Vinylphenol dimer | 766 | 1 | 0.02 | 4.044 |
| 2,3-Dihydroxybenzoic acid | 769 | 1 | <0.001 | <0.001 |
| Gentisic acid | 580 | 1 | 0.043 | 0.469 |
| Aminooxyacetic acid | 589 | 1 | 0.018 | 0.363 |
| 5-Hydroxytryptophan | 642 | 1 | 0.044 | 2.418 |
| Pentadecanoic acid | 806 | 1 | 0.039 | 3.125 |
| Sedoheptulose | 729 | 1 | 0.002 | 0.187 |
| D-Glyceric acid | 666 | 0.984 | 0.044 | 2.196 |
| Conduritol b epoxide | 799 | 0.984 | 0.047 | 2.492 |
| Benzoin | 673 | 0.953 | 0.033 | 2.647 |
| Gluconic lactone | 628 | 0.938 | 0.032 | 0.261 |
| Lyxose | 869 | 0.906 | 0.015 | 550.7 |
| 2-Monoolein | 817 | 0.906 | 0.036 | 4.227 |
| 2-Hydroxypyridine | 890 | 0.89 | 0.028 | 3.13 |
| 5-Methylresorcinol | 630 | 0.89 | 0.011 | 7.687 |
| 4-Hydroxyphenylacetic acid | 723 | 0.875 | 0.031 | 2.847 |
| Malonic acid | 195 | 0.875 | 0.003 | 0.29 |
| Allantoic acid | 793 | 0.859 | 0.048 | 4.021 |
| Stearic acid | 911 | 0.813 | 0.04 | 7.878 |
| Benzoic acid | 836 | 0.797 | 0.011 | 3.069 |
| 21-Hydroxypregnenolone | 668 | 0.766 | 0.038 | 3.557 |
| Caffeic acid | 679 | 0.75 | <0.001 | <0.001 |
| Galactinol | 670 | 0.703 | 0.002 | 1.919 |
| DL-Anabasine | 912 | 0.672 | 0.014 | 3.703 |
| Glucose-1-phosphate | 701 | 0.672 | 0.035 | 3.146 |
| Lactobionic Acid | 738 | 0.641 | 0.02 | 8.399 |
| Tyrosine | 554 | 0.625 | 0.02 | 2.836 |
| Carbamoyl-aspartic acid | 703 | 0.609 | 0.045 | 4.912 |

*^a^*AUC=area under the ROC curve.

*^b^*FC=fold change, mean value of peak area obtained from CS group / meanvalue of peak area obtained from AH group.


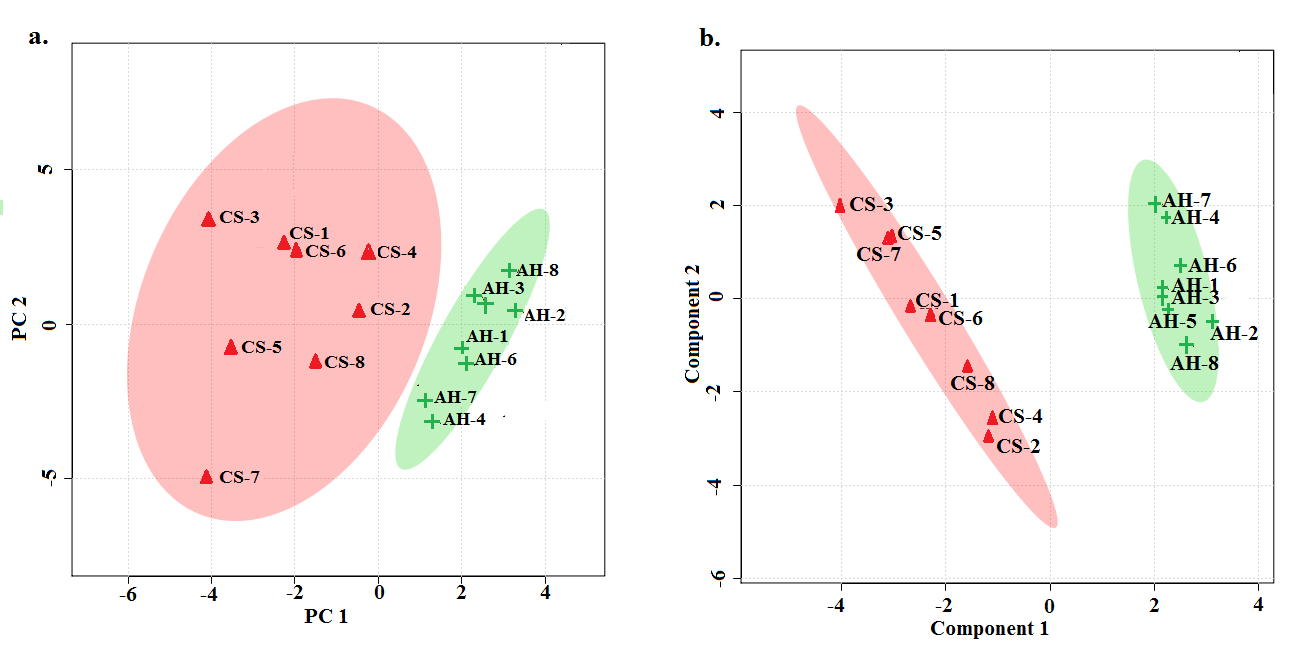


**Supplemental Figure S1.** The 2-D PCA score map (a) and 2-D PLS-DA score map (b) derived from the GC-TOF/MS metabolite profiles of urine for cows fed CS (red triangle) and AH (green plus). CS = dietcontaining corn stover as main forage; and AH = dietcontaining alfalfa and Chinese wild rye hay as main forage.
